# Supplementary material for: Pandemic-related experiences of older adults and people with disabilities
Source: PLoS One. 2025 Jun 17;20(6):e0325306. doi: 10.1371/journal.pone.0325306 (PMC12173368; doi:10.1371/journal.pone.0325306)
Supplement: Attachment — Submitted filename: PLOSONE_Response-to-Reviewers_30-04-2025.docx [file pone.0325306.s002.docx]

Response to Reviewers

| **Editor’s comments:** | Authors’ responses: |
| --- | --- |
| Thank-you for your patience with the review process. I think there are some final revisions to take into account to ensure theoretical and methodological clarity of your work. Both reviewers have made good suggestions and reviewer two did a very thorough read and picks up on the need to describe why these populations were chosen and some clarification of the sampling design. You do need to make a stronger case for the inclusion of the capabilities approach and justify that more. There is also a need to proofread which is normal and we hope you find their suggestions useful.  Please submit your revised manuscript by Apr 30 2025 11:59PM.  Please include the following items when submitting your revised manuscript: a rebuttal letter, a marked-up copy of your manuscript, an unmarked version of your revised paper without tracked changes. | Thank you for the comments and suggestions. The line numbers listed in our responses below are based on the marked-up copy of the manuscript. |
| If you would like to make changes to your financial disclosure, please include your updated statement in your cover letter. | No changes required to funding statement listed in cover letter. |
| Guidelines for resubmitting your figure files are available below the reviewer comments at the end of this letter. |  |
|  |  |
| When submitting your revision, we need you to address these additional requirements: |  |
| 1. Please ensure that your manuscript meets PLOS ONE's style requirements, including those for file naming. | Documents should now meet journal requirements. |
| 2. Thank you for stating in your Funding Statement: “This study did not receive any funding for the research, but it was supported by Dr. Ben Mortenson’s New Investigator Award from the Canadian Institutes of Health Research.”  Please provide an amended statement that declares *all* the funding or sources of support (whether external or internal to your organization) received during this study, as detailed online in our guide for authors at http://journals.plos.org/plosone/s/submit-now. Please also include the statement “There was no additional external funding received for this study.” in your updated Funding Statement. Please include your amended Funding Statement within your cover letter. We will change the online submission form on your behalf. | We can remove mention of Dr. Mortenson’s award so that the funding statement is consistent with the current one in cover letter. |
| 3. We note that the grant information you provided in the ‘Funding Information’ and ‘Financial Disclosure’ sections do not match.  When you resubmit, please ensure that you provide the correct grant numbers for the awards you received for your study in the ‘Funding Information’ section. | Statements are now consistent. |
| 4. In the online submission form, you indicated that [Anonymising qualitative interview transcripts does not make them fully unidentifiable, therefore data can be requested through Borealis (https://doi.org/10.5683/SP3/FOJDLJ) by researchers who meet the criteria for access to confidential data.]. All PLOS journals now require all data underlying the findings described in their manuscript to be freely available to other researchers, either 1. In a public repository, 2. Within the manuscript itself, or 3. Uploaded as supplementary information. This policy applies to all data except where public deposition would breach compliance with the protocol approved by your research ethics board. If your data cannot be made publicly available for ethical or legal reasons (e.g., public availability would compromise patient privacy), please explain your reasons on resubmission and your exemption request will be escalated for approval. | We have amended the statement in recognition of UBC’s ethical requirements: In accordance with our institution’s ethics policy, anonymising qualitative interview transcripts does not make them fully unidentifiable, therefore data can be requested through Borealis (https://doi.org/10.5683/SP3/FOJDLJ) by researchers who meet the criteria for access to confidential data. |
| 5. Please include your full ethics statement in the ‘Methods’ section of your manuscript file. In your statement, please include the full name of the IRB or ethics committee who approved or waived your study, as well as whether or not you obtained informed written or verbal consent. If consent was waived for your study, please include this information in your statement as well. | We have added the following statement to the methods section (line 159): Ethical approval was obtained from the University of British Columbia Behavioural Research Ethics Board and the Vancouver Coastal Health Research Institute (H20-01109). Prior to their initial interviews, participants emailed a signed copy of their consent form to the research team. |
| 6.  [Please include captions for your Supporting Information files at the end of your manuscript, and update any in-text citations to match accordingly. Please see our Supporting Information guidelines for more information: http://journals.plos.org/plosone/s/supporting-information.](https://www.editorialmanager.com/pone/%20Please%20include%20captions%20for%20your%20Supporting%20Information%20files%20at%20the%20end%20of%20your%20manuscript,%20and%20update%20any%20in-text%20citations%20to%20match%20accordingly.%20Please%20see%20our%20Supporting%20Information%20guidelines%20for%20more%20information:%20http:/journals.plos.org/plosone/s/supporting-information.%20) | N/A – the supporting document was only included because proof of ethical approval was previously requested. It should not be included in the documents related to any final publication. |
| 7. Please review your reference list to ensure that it is complete and correct. If you have cited papers that have been retracted, please include the rationale for doing so in the manuscript text, or remove these references and replace them with relevant current references. Any changes to the reference list should be mentioned in the rebuttal letter that accompanies your revised manuscript. If you need to cite a retracted article, indicate the article’s retracted status in the References list and also include a citation and full reference for the retraction notice. | We noted one author’s name was incorrectly listed so we have amended this (ref. 23). |
|  |  |
| **Reviewers' comments:** |  |
| Reviewer #1: |  |
| This manuscript was a revised version of already assessed by 3 reviewers. The authors certainly reply effectively to all comments provided in the appendix my only comments are as follow: |  |
| 1) typo line 43 - recommendation with a s | We have changed it to recommendations (line 49). |
| 2) line 79 - The authors: can they clarify if it was another team a sit is difficulty to understand who they are | We changed “the authors” to read “we” to avoid confusion (line 90). |
| 3) line 95 and 98 please change the word things as it is very vague and do not bring any value or understanding of the useful content | We have amended text to make it clearer (line 109). |
| 4) line 150 add (BC) | We have added (BC) after British Columbia (line 167). |
| 5) line 525 - change 344 to 34 | Thank you, we have changed 344 to 44 (line 555). |
| 6) great aspect: the authors use evidence based material from previous published studies | Thank you. |
| 7) rigorous method for data analysis |  |
|  |  |
| Reviewer #2: |  |
| The manuscript describes the experiences of older adults and people with disabilities during the first few months of the COVID-19 pandemic. The authors describe Sen’s Capability approach and how it was used in their qualitative thematic analysis. There are some very impactful quotes and discussion points, however significant revisions are needed. |  |
| Major comments: |  |
| There is poor connection as to why these 4 populations were pooled in this publication, as there is little justification as to why these groups are being considered together. How do older adults without disability relate to people with disabilities? It seems like very disparate data and OA is being conflated with disability without justification as to why. This is an issue from the introduction and continues with little linkage or explanation between OA and the other groups in the results and discussion. | We have added statements indicating the connection and relevance of exploring older adults and various disabilities together (lines 66, 86, 93). The two core elements are the similarities in physiological characteristics (e.g., risk of infection, mobility limitations, mortality), and the associated forms of discrimination and how they are experienced (i.e., ableism and ageism). |
| For the population group of other disabilities, was there some recruitment strategy for individuals with physical, cognitive, or other disabilities? Strongly suggest clarifying what types of disabilities are/could be includes here would add great value. The disability community is very heterogenous and some description would allow for a better understanding of capabilities/inequities this group experience that are distinct from the stroke or SCI groups. In results there is some of this description attached to quotes but there needs to be a cohesive description earlier. This also ties into the previous comment about the lack of connection between study groups. | Our recruitment strategies following convenience, but we recruited a range of disabilities (physical, mental, sensory, cognitive). We have listed groups contacted from previous research (line 174), and provided a breakdown of the disabilities ultimately included (line 184). These are now consistent with descriptions in the results, and we have added any descriptions which were missing in the results. |
| The addition of Mitra, Trani et al, etc. based on other reviewer comments does not add value as currently written. As there is no discussion of how/why the authors consider this in their study (line 134). Perhaps this could be further explored in methods (lines 206-20) where capability approach is mentioned (but its use could also be further detailed). | We have added a sentence at the end of this discussion to try and address this concern. “This suggests the need to consider how capabilities should be supported on a community level to enable everyone to have the same opportunities (22).” |
| Overall, a careful round of editing is needed as there are syntax and punctuation issues, some noted below. | Thank you for noting some below, we have addressed these along with other outstanding issues. |
|  |  |
| Minor comments: |  |
| General: In some cases oxford comma is used and other times is not, suggest being consistent. | We have now used the oxford comma consistently throughout. |
| Introduction: lines 57-59 read as though people with disabilities wish to receive higher priority. Whereas their concern lies more with medical ableism and not being fairly assessed based on quality of life bias. Suggest re-phrasing to emphasize the validity of their concern. | Thanks for pointing that out. We have changed the text to indicate: “In the ~~absence of adequate intensive care beds and respirators~~ face of entrenched ableism…” (line 64). |
| Lines 69-71 needs editing to clarify. | We have edited this sentence (line 76). |
| Line 98 check comma placement between “broader” and “social”. | The comma between broader and social has been removed (line 112). |
| Line 109 “lives in place” suggest change to “lives in a place” | We have changed to lives in a place (line 123). |
| Lines 120-123 presents Mirta’s model with and without capitalizations. | Capitalisation has now been used consistently (line 133). |
| Methods: Line 142 used involved twice, would suggest other word choice. | We changed “which involved” to “and subsequent” (line 156). |
| Line 145 suggest adding ethic review #s | We have added the ethics number (line 161). |
| Lines 162,166 repeats the same “The majority of participants were born in Canada”. | We have removed the second occurrence. |
| Lines 189-190 were the interviewers also the coders? IE. Did people use their lived/living experience of disability to inform the analysis? You reference positionality of interviewers but it is not clear for the rest of the research process (lines 211-213). | We have provided this additional information: “Interviewers and additional team members conducted a thematic analysis of the data” (line 208).  So some people with lived experience were involved in the analysis process. |
| Lines 189-207 Needs clarification if there were different codes for each of the 4 populations (IE 4 different coding guides were made) or 1 coding book based on all 4 populations. A flowchart to illustrate this process could be a good addition. | Independent analyses were conducted for each sub-sample. Researchers then collaborated to develop a coding scheme for the data set as a whole.  We have made this clearer in the methods (line 216). |
| This may help you for describing processes of qualitative data integration as well as how you used Sen’s framework in your methods: DiMartino, L., Carroll, A.J., Ridgeway, J.L. et al. Development of a method for qualitative data integration to advance implementation science within research consortia. Implement Sci Commun 6, 21 (2025). https://doi.org/10.1186/s43058-025-00701-4 | Thanks for this citation. Unfortunately the focus of this work seems on integrating the findings of different studies across different research sites, where we had a single study (albeit with different sub-samples) at one site. |
| Results: Line 216-220 punctuation and syntax issues, including multiple colons in one sentence. | We have addressed the punctuation and syntax issues (line 240). |
| Line 244 missing period | We have added a period (line 270). |
| Line 342 “the challenges the experienced” should be “the challenges they experiences” | We changed it to the challenges they experienced (line 370). |
| Line 353 “a physically health life” should be “healthy” | We changed it to healthy (line 381). |
| Line 374 In quote - Girl Guide lades – would this not be ladies? Check for typo or note sic? | We changed it to ladies (line 402). |
| Line 457-462 Quote about appreciation of healthcare workers is not clear how it links to the sub-theme of “wanting to exert control” | The full title of this sub-theme is “Wanting to exert control in the face of constrained capabilities,” so this quote illustrates how some participants chose to react in a positive way, through the expression of gratitude, in the face of these restrictions.  We have altered the way that quote is introduced to better convey that idea (line 486): Despite the restrictions imposed upon them, some participants chose to express gratitude towards the frontline workers in healthcare and decision makers, as.  a 76-year-old female (OA 2) indicated: |
| Discussion: Line 524 citation # not correct | This has been changed to 44 (line 555). |
| Line 527 “increased disability” should be changed. This is equating health with disability, which does not align with how you describe disability in lines 64-65 as health consequences or how you describe it in the Human Development Model. | We have replaced “increased disability” with further complications and decreased function (line 557). |
| Line 547 – “without abilities” should be disabilities? | We have changed it to disabilities (578). |
| Limitations: Line 551- You describe COVID-19 as an epidemic here, but pandemic elsewhere. | We have changed it to pandemic (584). |
|  |  |
| *In compliance with data protection regulations, you may request that we remove your personal registration details at any time.* [*(Remove my information/details)*](https://www.editorialmanager.com/pone/login.asp?a=r)*. Please contact the publication office if you have any questions.* |  |
